# Editorial: Advances in Endocrinology: Stem Cells and Growth Factors

**DOI:** 10.3389/fendo.2020.00564

**Published:** 2020-08-14

**Authors:** Andrea Ballini, Salvatore Scacco, Rajiv Saini, Stefania Cantore, Giorgio Mori

**Affiliations:** ^1^Department of Biosciences, Biotechnologies and Biopharmaceutics, University of Bari “Aldo Moro, ” Bari, Italy; ^2^Department of Precision Medicine, University of Campania “Luigi Vanvitelli, ” Naples, Italy; ^3^Department of Basic Medical Sciences, Neurosciences and Sense Organs, University of Bari “Aldo Moro, ” Bari, Italy; ^4^Faculty of Dentistry, University of Medicine, Tirana, Albania; ^5^Department of Sports Sciences, City Unity College Athens, Athens, Greece; ^6^Department of Clinical and Experimental Medicine, University of Foggia, Foggia, Italy

**Keywords:** stem cells research and therapy, translational endocrinology, cell biology, growth factors, diseases

Stem Cells investigation in Endocrinology: leading stem cell scientists and developmental endocrinologists, critically review both cutting-edge approaches to stem cell biology and the application of stem cells and their secretome to translational/precision medicine, endocrine diseases, including diabetes, tissue/organ repairs, energy metabolism, and metabolic disorders (1–4).

Current research trends in Endocrinology are directed to modify stem cells, develop an endocrine-like cell, and use adult mesenchymal stem cells (MSCs) to treat autoimmune diseases, including endocrine-based autoimmune diseases (5).

Several growth factor ligand and receptor gene products have been shown to play roles during wound healing in endocrine-related diseases such as foot ulcers that affect a range of patients with diabetes, resulting in a great health burden (6).

During recent years, along with advances in the biomedical sciences, various surgical and non-surgical therapeutic methods have been suggested to promote wound healing; growth factors involved in these processes, which could facilitate tissue regeneration in patients with diabetes are currently widely investigated, including epidermal growth factor, vascular endothelial growth factor, transforming growth factor-beta, fibroblast growth factor, platelet concentrates and erythropoietin (7–10).

The articles in this topic highlight the effect of stem cells, their secretome, within the context of isolation, derivation, reprogramming, self-renewal, quality control, feasibility, as well new methodological paradigms that challenge current thinking in clinical research, including differentiation, transplantation and “proof-of-concept” studies in translational endocrinology.

In detail, Human fetal stem cells (HFCs) are valuable source for cell therapy and co-transplantation of MSCs can improve therapeutic effects of both stem cells. A research article provided in this special issue by Arjmand et al., on Type 1 diabetic mice model, suggested that co-transplantation of MSCs and HFCs enhance overall stem cells engraftment and subsequently their therapeutic effects in various diseases including Type 1 diabetes.

In a review, the same group with further authors, discuss the recent researches, features and advantages of zebrafish that can have a leading role as a promising animal model, for personalized regenerative diseases studies, as also in endocrine diseases, providing a good perception of underlying mechanism.

In this special issue, Kulebyakin et al., draw reader's attention to evolutionary changes that occurred in growth factors and their receptor tyrosine kinases (RTKs) and how they established and shaped response to injury in metazoan with an evolutionary rational. Their communication invites the reader to re-evaluate functions of growth factors as keepers of natively existing communications between elements of tissue which makes them a fundamental component of successful regenerative strategy.

Among the many hypotheses formulated on the aetiopathology of an endocrinological disease such as obesity, a key role, as reported from Di Domenico et al., can be attributed to the relationship between stress oxidative and intestinal microbiota. As stated from the authors, obesity acts negatively on the wound healing, and multiple evidences tend to show that genetic, epigenetic, and lifestyle factors contribute to determine in the obese an imbalance of the redox balance correlated with the alteration of the intestinal microbial flora.

Moreover, in an additional review, Arjmand et al., point MSCs as pivotal candidates for regenerative medicine purposes and, especially for bone regeneration, are described as the most common type of cells with anti-inflammatory, immune-privileged potential, and less ethical concerns than other types of stem cells which are investigated in osteoporosis. Based on several findings, the MSCS effectiveness near to a great extent depends on their secretory function. Indeed, they can be involved in the establishment of normal bone remodeling via initiation of specific molecular signaling pathways.

Finally, as stead from Tatullo et al., in this scenario, the identification of predictive markers becomes a significant diagnostic and prognostic factor, other than, it would favor the use of certain drugs in a specific patient population.

In conclusion, all the articles dispensed an update on the latest evidence regarding the use of stem cells and growth factors in Endocrinology, thus identifying possible alternative therapeutic targets.

## Author Contributions

All authors listed have made a substantial, direct and intellectual contribution to the work, and approved it for publication.

## Conflict of Interest

The authors declare that the research was conducted in the absence of any commercial or financial relationships that could be construed as a potential conflict of interest.
